# Mechanisms regulating vascular and lymphatic regeneration in the heart after myocardial infarction

**DOI:** 10.1002/path.6093

**Published:** 2023-06-05

**Authors:** Bronwyn Berkeley, Michelle Nga Huen Tang, Mairi Brittan

**Affiliations:** ^1^ Centre for Cardiovascular Science, The Queen's Medical Research Institute University of Edinburgh Edinburgh UK

**Keywords:** myocardial infarction, neovascularisation, lymphangiogenesis, endothelial cells, VEGF, cardiac regeneration, heart failure

## Abstract

Myocardial infarction, caused by a thrombus or coronary vascular occlusion, leads to irreversible ischaemic injury. Advances in early reperfusion strategies have significantly reduced short‐term mortality after myocardial infarction. However, survivors have an increased risk of developing heart failure, which confers a high risk of death at 1 year. The capacity of the injured neonatal mammalian heart to regenerate has stimulated extensive research into whether recapitulation of developmental regeneration programmes may be beneficial in adult cardiovascular disease. Restoration of functional blood and lymphatic vascular networks in the infarct and border regions via neovascularisation and lymphangiogenesis, respectively, is a key requirement to facilitate myocardial regeneration. An improved understanding of the endogenous mechanisms regulating coronary vascular and lymphatic expansion and function in development and in adult patients after myocardial infarction may inform future therapeutic strategies and improve translation from pre‐clinical studies. In this review, we explore the underpinning research and key findings in the field of cardiovascular regeneration, with a focus on neovascularisation and lymphangiogenesis, and discuss the outcomes of therapeutic strategies employed to date. © 2023 The Authors. *The Journal of Pathology* published by John Wiley & Sons Ltd on behalf of The Pathological Society of Great Britain and Ireland.

## Introduction

Heart failure (HF) affects ~1–3% of the global adult population [1], with 64.3 million people affected worldwide in 2017 [2]. Patients with HF have a poor prognosis, with an estimated mortality of up to 60% by 5 years [3]. Myocardial infarction (MI) is the leading cause of HF and a history of MI increases the risk of developing HF by 28% [4]. Early reperfusion by primary percutaneous coronary intervention (PCI) after acute MI can limit infarct size [5] and has significantly improved survival rates and complications of acute MI. However, patients with no‐reflow phenomenon (myocardial hypoperfusion despite seemingly successful PCI), and those who present late after MI with extensive injury are at greater risk of adverse outcomes including HF [6, 7]. Current pharmacologic treatments for HF can delay disease progression but cannot ultimately halt or reverse fibrosis and adverse cardiac remodelling, and thus are non‐curative [8].

New approaches are urgently required to enhance myocardial perfusion, limit infarct expansion, and promote cardiac regeneration after MI. While the field of cardiovascular regeneration has focused much of its efforts on remuscularisation [9], a central requisite for cardiac regeneration is the rapid and effective restoration of functional blood vascular and lymphatic networks to support and nourish the myocardium and promote survival, repair, and regeneration pathways [10, 11]. Therefore, a comprehensive understanding of the intrinsic mechanisms that underpin coronary blood and lymphatic growth during cardiac remodelling after MI may inform the next generation of cardiovascular regenerative strategies.

## Neonatal and developmental cardiac regeneration

Cardiac regeneration after injury was first reported in the 1970s and was long thought to be restricted to amphibians and fish [12, 13, 14]. However, neonatal mammalian heart regeneration was later shown in mice [15, 16] and pigs [17, 18], although this regenerative capacity was lost within a week after birth. Early neonatal mouse myocardial regeneration studies focused predominantly on the mechanisms governing cardiomyocyte migration and proliferation after injury [16]. However, later studies revealed that endothelial cell (EC) activation and proliferation was a critical step during neonatal mouse heart regeneration, and that it preceded cardiomyocyte renewal, as evidenced by the extensive sprouting of collateral arteries at 4 days post‐MI via CXCR4 and CXCL12 signalling [19].

## Cardiac regeneration in humans

Complete functional cardiac recovery was observed in a human newborn patient with severe MI due to coronary artery occlusion. After a 12‐month follow‐up, the patient's heart was shown to be indistinguishable in function and morphology compared with the hearts of age‐matched healthy patients [20]. This indicates that, similar to fish and neonatal mammals, newborn humans may have endogenous capacity to repair myocardial damage and recover cardiac function after MI.

Although the adult human heart was long considered a post‐mitotic organ, cardiomyocyte proliferation throughout the adult human lifespan was reported in a unique study by Bergmann *et al* [21]. Carbon‐14 concentrations were measured in cardiomyocyte DNA from individuals born before and after nuclear bomb tests during the Cold War [21]. The same group later reported that adult human cardiac ECs have proportionately high proliferation rates (>15% per year), compared with cardiomyocytes and mesenchymal cells (<4% per year) [22]. However, these studies were met with controversy, based on concerns about the methodology, interpretation of data, and appropriateness of patient samples [23], and the findings remain to be replicated.

In summary, despite some evidence that pathways for cardiac regeneration may be preserved in the adult human heart, these are clearly insufficient to support physiological recovery following ischaemic injury. It is likely that, similar to other mammalian species, the potency for functional cardiac regeneration in humans is restricted to early development.

## Structure and function of lymphatic and blood vascular systems

Vertebrates have two circulatory systems: the blood and lymphatic vasculatures, functioning as the main supply and drainage systems of the body. The blood vasculature transports solutes, fluid, macromolecules, hormones, and circulating cells through closed pulmonary and systemic circuits [24]. The lymphatic vasculature system maintains interstitial fluid homeostasis, transports haematopoietic cells for immune surveillance, and absorbs dietary lipids from the gastrointestinal tract, through a complimentary unidirectional open circulatory system [24].

The vascular endothelium is a monolayer of ECs that constitutes the inner lining of arteries, veins, and capillaries. Vascular ECs (VECs) have numerous endocrine functions. Other than acting as a barrier between blood and tissues, the vascular endothelium regulates vascular relaxation and constriction. VECs are important in controlling blood fluidity, platelet adhesion and aggregation, leukocyte activation, adhesion, and transmigration. VECs also precisely regulate the balance between coagulation and fibrinolysis. VECs play a key role in regulating immune response and inflammation. They direct inflammatory cells to pathogens and wounded areas in need of defence/repair. Normally quiescent, local ECs become activated upon tissue injury/ischaemia to allow vessel sprouting in a sequential process involving basement membrane degradation, EC detachment, migration and proliferation, vessel fusion, and maturation [25].

Lymphatic vessels are constructed from three components: initial lymphatics, pre‐collector lymphatics, and collector lymphatics. Lymphatic ECs (LECs) form a monolayer to line the lymphatic vasculature connected by button‐like junctions. Lymphatic capillary LECs are attached to the surrounding extracellular matrix (ECM) with anchoring filaments, unlike blood VECs that are attached to the basement membrane. Increased interstitial pressure, as in oedema, distorts the ECM components on these anchoring filaments and enables increased permeability of lymphatic capillaries to enhance drainage of excess extravasated fluid. Larger pre‐collector and collector vessels have some smooth muscle cell coverage with a continuous basement membrane. The lymphatic system modulates immune response by trafficking antigens, pathogens, and immune cells from sites of inflammation and infection to regional lymph nodes. There is an influx of interstitial fluid and immune cells during injury and inflammation that necessitates an expansion of lymphatic vasculature.

In the context of MI, lymphatic and blood vascular ECs play a critical role in the resolution of inflammation and trafficking of immune cells. Tissue repair after MI involves coordinated robust angiogenic and lymphangiogenic responses to resolve the necrotic infarct core and reduce myocardial dysfunction [26].

## Neovascularisation and lymphangiogenesis in the heart

Neovascularisation is the umbrella term that refers to the growth of new vascular networks through *de novo* sprouting from migrating progenitor cells (vasculogenesis) and via the expansion of pre‐existing blood vessels (angiogenesis) [27, 28]. Arteriogenesis is the growth and enlargement of pre‐existent collateral arterioles initiated by elevated shear stress in the vessel wall [29, 30, 31]. To achieve complete cardiac regeneration, the reconstruction of an efficient vascular network is crucial to supply regenerating cardiomyocytes with oxygen and nutrients, as well as to eliminate metabolic products [32].

Lymphangiogenesis, the formation of new lymphatic capillaries, occurs in the infarct region and extends into the subepicardium of non‐infarcted areas [26]. MI causes increased interstitial fluid accumulation, resulting in myocardial oedema [33, 34]. Myocardial inflammation can also cause rarefaction and dysfunction of blood vasculature [35] and of pre‐collector and collector lymphatic vessels, which may influence immune cell clearance and promote oedema [26, 36]. However, myocardial oedema can persist for up to 6–12 months post‐MI in humans, suggesting that, similar to other endogenous mechanisms of cardiovascular regeneration, innate lymphangiogenesis pathways are inadequate to prevent lymphatic insufficiency [37]. In summary, endogenous mechanisms of neovascularisation and lymphangiogenesis are pertinent targets for therapeutic strategies post‐MI but are complex and remain to be further understood.

## Temporal angiogenic and lymphangiogenic responses after MI

**Figure 1:**
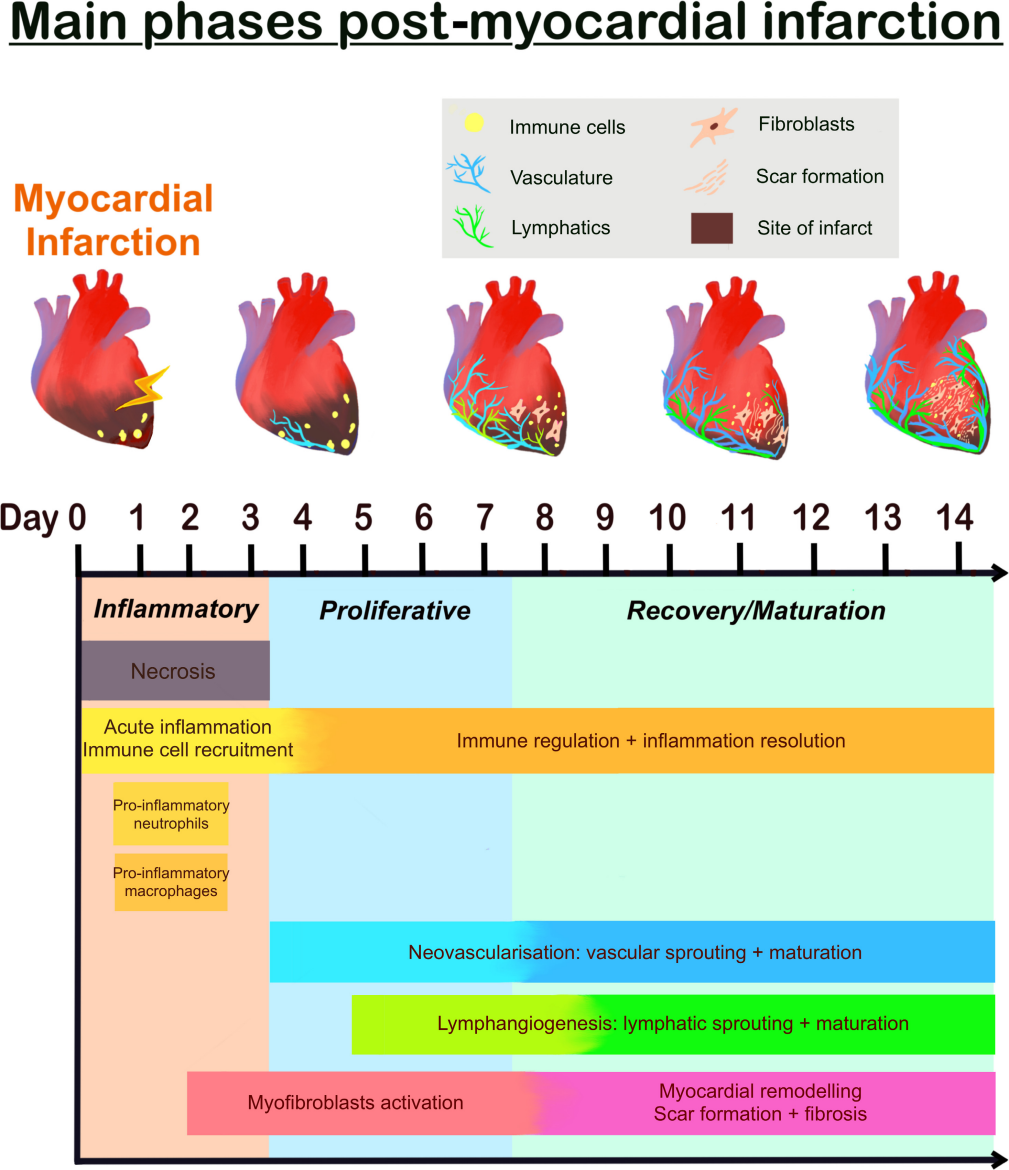
Schematic overview of the temporal cardiac regeneration in mice after myocardial infarction. Top panel indicates the key for the schematic and graphical representation of injured/regenerating heart in mice.

The endogenous responses in the heart after MI are a complex and finely timed interplay spanning inflammation to fibrosis, that are often compartmentalised into distinct phases in mice over the course of several days. Importantly, each phase involves a concerted effort by several cell types within the heart, including epicardial cells, ECs, nerves, fibroblasts, myofibroblasts, and lymphatic cells [38]. In brief, initial ischaemic injury and cardiomyocyte necrosis are accompanied by an extensive inflammatory response phase. This phase involves the migration and accumulation of macrophages and monocytes into the infarct to clear damaged cells and ECM components. This is followed by a proliferative or reparative phase where inflammation is resolved, neovascularisation pathways are activated, and reparative myocardial remodelling is initiated via (myo)fibroblast proliferation. During the recovery phase, activated fibroblasts and myofibroblasts continue to mediate scar formation and fibrosis [39] (Figure 1).

After MI, heart regeneration relies on rapid neovascularisation to guide cardiomyocytes to the area of injury and support myocardial regrowth. New capillary growth was found in the apical thrombus as early as 2 days post‐apical resection in neonatal mice [40]. The development of mature arteries and perfused vessels appeared by 5 days post‐resection [40]. Of note, vessel ingrowth preceded cardiomyocyte migration, with co‐alignment of most migrating cardiomyocytes with ingrowing vessels [40]. Enhanced angiogenesis can rescue MI‐induced damage in the myocardium by mitigating hypoxia in the ischaemic border zone. In adult mice, primitive vessels with different diameters were observed 3 days post‐MI and extended from the endocardium [41]. Between 4 and 14 days post‐MI, primitive vessels were covered by pericytes and became a mature circulatory network with uniform vessel diameters. This salvaged the damaged cardiomyocytes via activating vascular endothelial growth factor (VEGF) receptor 2 (VEGFR2) signalling [41]. Similarly, it was reported that arterial ECs in the neonatal mouse heart at 4 days post‐MI had reassembled, formed collateral arteries, and provided an alternate route to vessel perfusion [19]. However, artery reassembly did not occur in injured P7 or adult murine hearts. Hence, this suggested that artery‐derived collateral formation was restricted to a brief regenerative window [19]. Investigating the role of early collateral blood flow in MI patients showed that the presence of well‐developed collateralisation was associated with reduced infarct size and improved myocardial salvage [42]. This highlights the cardioprotective role of endogenous neovascularisation after MI.

Fibrotic pathways also play an important role in the adult mammalian heart after ischaemic injury. By 3 days post‐MI, a substantial number of cardiac fibroblasts were shown to undergo mesenchymal‐to‐endothelial transition via the p53 pathway, to promote neovascularization and cardiac repair [43]. In contrast, other studies have demonstrated that most cardiac fibroblasts maintain their phenotype after injury to mediate fibrosis [44, 45]. Lineage tracing in adult murine hearts showed that cardiac fibroblasts expanded after injury but did not contribute to neovascularisation. Instead, the development of new vessels was almost exclusively derived from pre‐existing ECs [27]. Complementary studies using endothelial‐specific lineage tracing ‘Confetti’ mice showed that endogenous vascular repair following MI was maintained via clonal proliferation of pre‐existing resident ECs [46, 47]. No significant contribution from bone marrow cells or endothelial‐to‐mesenchymal transition to new blood vessels was observed at day 7 post‐MI, shown using single‐cell RNA‐sequencing (scRNA‐seq) technology [47]. However, a population of ECs undergo transient mesenchymal differentiation to facilitate neovascularisation at 14 days after injury [46]. This suggests that endothelial‐to‐mesenchymal transition may be temporally regulated during different stages post‐MI. Potentially, this transient mesenchymal activation after injury may facilitate EC migration and clonal expansion to revascularise the human heart.

Three key studies have contributed to our understanding of the temporal dynamics of mammalian cardiac lymphangiogenesis in mice post‐MI [48, 49, 50]. An increase in lymphatic vessel density at 7 days post‐MI was observed at the surface of the heart that increased in diameter by day 14 and continued to expand until day 35 post‐MI, where lymphatic shunts were apparent at the border zone of the infarct and healthy myocardium [48]. Similarly, increased lymphatic vasculature at 4 and 8 days post‐MI was reported, which remained elevated at 42 days post‐MI compared with healthy mice [49]. A significant increase is seen in the total number of LECs and of proliferating LECs at 3 days post‐MI, which persisted until day 7 following injury [50]. Investigations of the remodelling of cardiac lymphatics reported an ‘explosion’ of lymphatic density in the infarct scar by 12 weeks post‐MI in comparison with sham‐operated rats [26]. In zebrafish, cardiac lymphatic vessel growth appeared as early as 40 h post‐injury, with significant expansion observed at 7 days after injury [51, 52]. These studies collectively show expansion of lymphatic vessels at early stages after MI, which can persist for prolonged periods, thereby indicating a likely regulatory role in the regenerative process.

Histopathological analyses have been used to study lymphangiogenesis in myocardial remodelling in autopsied hearts from MI patients [53]. The authors used the Lodge‐Patch scale [54] to characterise 88 lesions, spanning all seven stages of the scale (Table 1). Importantly, they found that lymphangiogenesis precedes angiogenesis post‐MI, and that significant angiogenesis was observed between stages III and IV, during which cardiomyocyte necrosis was observed. Similarly, in the rat mesentery, angiogenesis was found to precede lymphangiogenesis after an inflammatory stimulus [55]. Furthermore, the angiogenic effect of VEGF‐C was attenuated in the presence of an expanding lymphatic network in the rat mesentery [56]. In particular, endothelial proliferation and the number of branch points in the blood vasculature were reduced. These studies suggest a regulated temporal relationship between neovascularisation and lymphangiogenesis pathways following MI. Whether strategies to augment blood vascular and lymphatic responses by targeting common regulatory mechanisms support myocardial regeneration remains to be determined. However, it is imperative to establish the molecular orchestration of these events to inform future strategies.

**Table 1:** The seven stages of histopathological change after MI – Lodge‐Patch scale.

Stage	Description
I	Earliest changes where stretching and waviness of myocardial fibres are observed and these myocytes have eosinophilic cytoplasm and pyknosis
II	Coagulation necrosis of cardiomyocytes with haemorrhage or neutrophil infiltration, but without CD68^+^ macrophages
III	Coagulation necrosis of cardiomyocytes with infiltration of CD68^+^ macrophages in addition to neutrophils
IV	Early stage of granulation in which fragmented myocytes with coagulation necrosis, many CD68^+^ macrophages, a few neutrophils, and fibroblasts are found
V	Mature granulation tissue with CD68^+^ macrophages and fibroblasts, but without necrotic myocytes or neutrophils
VI	The stage of fibrosis with abundant myofibroblasts positive for smooth muscle actin, in which the interstitium is weakly stained blue by Azan–Mallory throughout the lesion
VII	The lesion is replaced with scar tissue with a decrease of myofibroblasts and uniformly stained blue by Azan–Mallory

## Pre‐clinical studies of neovascularisation in the heart after MI

Following MI, most processes regulating the activation of vessel growth and vascular remodelling are impaired by the deleterious microenvironment characterised by fibrosis, inflammation, hypoperfusion, and inhibition of angiogenic and regenerative programmes [57]. Thus, targeting vascular homeostasis (e.g. hypoxia‐related pathways, immune‐inflammatory balance, and haemodynamic forces) and stimulating neovascularisation could be appropriate for the restoration of functional vascular networks in the ischaemic heart [57, 58].

Since endogenous pathways in the adult heart cannot support cardiac regeneration after MI alone, there is a rationale to bolster intrinsic neovasculogenic signals through the administration of exogenous pro‐angiogenic factors (i.e. growth factors, microRNAs, modified RNA, exosomes, proteins) [8, 59, 60, 61]. Indeed, several proof‐of‐concept pre‐clinical studies have shown the potential benefit of this strategy. For example, intramyocardial injection of synthetic modified RNA encoding human VEGF‐A stimulated expansion of epicardial cells and directed their differentiation towards an endothelial lineage [62]. This resulted in enhanced myocardial perfusion and improved survival in a mouse MI model [62]. Lentiviral ETV2 (ETS variant transcription factor 2) delivery into murine infarcted hearts upregulated the expression of pro‐angiogenic, anti‐fibrosis, and anti‐inflammatory factors [61]. A novel population of cardiac interstitial cells named telocytes was shown to facilitate cardiac angiogenesis and regeneration after MI by inhibiting the apoptosis of cardiac microvascular ECs [63]. Cardiac telocyte‐derived exosomes targeted and silenced the cell death inducing p53 target 1 (*Cdip1*) gene, thus reducing activated caspase‐3 [63]. It was suggested that the promotion of EC survival and suppression of apoptosis could aid long‐term therapeutic cardiac angiogenesis. Five‐week treatment with thymosin‐β4 in mice subjected to MI prevented cardiac rupture and improved cardiac function with significantly ameliorated left ventricle dilation, reduced cardiac fibrosis, and an enhanced capillary density/angiogenic response [64]. Adeno‐associated virus (AAV) 9‐mediated delivery of the transcription factor zinc finger E‐box‐binding homeobox 2 (*Zeb2*) in the infarcted murine heart induced the release of pro‐angiogenic factors, thymosin‐β4 and prothymosin, and contributed to improved cardiac repair and function by stimulating angiogenesis [65]. These findings revealed beneficial mediators of angiogenesis which may offer potential therapeutic opportunities for patients with MI.

## Reactivation of developmental gene programmes as a therapeutic approach in adult cardiovascular disease

The hypothesis that genes expressed during embryogenesis may be reactivated and repurposed to initiate tissue regeneration is gaining traction [66]. Indeed, in the adult zebrafish heart, embryonic epicardial genes, including *raldh2* and *tbx18*, were shown to be reactivated following injury and promoted cardiomyocyte proliferation and myocardial regeneration [67, 68, 69]. Developmentally activated epicardial‐derived cells facilitated regeneration by invading and revascularising the new myocardium via fibroblast growth factor (*Fgf*) signalling [68]. Injury‐stimulated epicardium and endocardium contributed to cardiac regeneration by providing guidance cues and VEGF‐A signalling to induce coronary revascularisation and provide a scaffold to support cardiomyocyte replenishment [51, 70].

Foetal gene reactivation in heart injury and disease has also been shown to occur in other cardiac cell types [71]. Wilms’ tumour 1 (*Wt1*), an essential gene for normal heart development during embryogenesis, is activated in the coronary vasculature after cardiac damage in adult zebrafish [72] and rats [73]. The epicardium is normally quiescent in the murine adult heart, but in response to injury stimuli, quiescent lineages can be reactivated to re‐express embryonic developmental genes, i.e. *Wt1*, T‐box factor 18 (*Tbx18*), transcription factor 21 (*Tcf21*), and retinoic acid‐synthesising enzyme (*Raldh2*), in an attempt to repair and revascularise the ischaemic heart [74, 75, 76]. The injury‐stimulated adult mouse epicardium recapitulated foetal epicardial properties and promoted proliferation of epicardium‐derived cells from 3 days to 2 weeks after MI, which then differentiated into mesenchymal cells. These cells modulated myocardial injury and supported angiogenesis by secreting paracrine factors [77]. One study showed that *de novo* capillary network formation in the infarct border zone and necrotic infarct core after MI in adult mice was formed by cells originating in the endocardium and coronary sinus. Thus, the reactivated epicardium appears to support neovascularisation by directionally promoting outgrowth of vessels toward the infarcted zone [78]. Together, these studies highlight the exciting potential of reactivation of foetal genes in the adult heart after MI, and improved in‐depth knowledge will be crucial to inform new therapeutic strategies.

Importantly, care must be taken. An aberrant expression of foetal genes has been linked to maladaptive changes in cardiac function in the adult failing heart. The ventricular re‐expression of some foetal genes, including atrial and brain natriuretic peptide, isoforms of contractile proteins, foetal‐type cardiac ion channels, and some smooth muscle genes, is thought to be associated with cardiac remodelling in response to pathological stress [79]. Indeed, foetal gene programmes are frequently used as biomarkers of cardiac hypertrophy and HF in pre‐clinical models [80]. In addition, several foetal genes, e.g. limb‐bud and heart (*Lbh*), frizzled receptor‐2 (*Fzd2*), fibulin‐1 (*Fbln1*), and tenascin C (*Tnc*), were identified to be reactivated in cardiac ECs during myocardial remodelling and HF [81]. It is highly feasible that the re‐expression of developmental genes in adults requires precise timing and regulation, i.e. if sustained or dysregulated it may progress to pathological changes [82]. Interestingly, the reparative process in adult hearts is correlated with high and robust expression of inflammatory and fibrotic genes, whereas the injured foetal heart demonstrated diminished inflammatory and fibrotic responses leading to complete cardiac regeneration [83]. Therefore, future research should focus on temporal regulation of reactivated developmental genes, as well as their capacity to minimise cardiac remodelling and fibrosis.

## Pre‐clinical studies for lymphangiogenesis in the heart after MI

The VEGF family members are key regulators of angiogenesis and lymphangiogenesis [56, 84]. VEGF‐C and VEGF‐D are increased in the infarcted heart at both early and late stages of MI in humans [85]. They are known to drive endogenous lymphangiogenic responses post‐MI by promoting lymphatic capillary expansion. In addition, low VEGF‐C is an independent predictor of all‐cause mortality in patients with suspected or known coronary artery disease [86]. Therefore, VEGF‐C has been a key focus of lymphangiogenesis studies after MI.

Several rodent studies have reported a therapeutic benefit of VEGFR3‐selective VEGF‐C gene or protein therapy to drive lymphangiogenesis and improve cardiac function post‐MI. One study utilised an LEC‐specific VEGF‐C, VEGF‐C_156S_, which acts specifically via the VEGFR2 receptor, and augmented the lymphangiogenic response to MI and promoted immune cell clearance [48]. Notably, left ventricular ejection fraction (LVEF) was improved 14 and 21 days post‐MI following VEGF‐C_156S_ treatment. Therapeutic VEGF‐C improves clearance of acute inflammation by trafficking immune cells towards draining mediastinal lymph nodes [11]. Delivery of a VEGFR‐3 selective ligand, VEGF‐C_C152S_, in a rat MI model showed reduced cardiac hypertrophy and attenuation of pre‐collector remodelling [26]. Studies utilising AAV delivery of *Vegfc* or intramyocardial VEGF‐C delivery did not report effects on infarct size or significant functional improvement [26, 87], although lymphangiogenesis and angiogenesis were not quantified in these studies.

A recent study challenged the impact of VEGF‐C on coronary lymphangiogenesis [88]. The effects of lymphatic or vascular EC‐specific loss of VEGFR3, and global loss of VEGF‐C and VEGF‐D ligands on cardiac function post‐MI in mice were studied. LVEF 2 weeks post‐MI was not impaired by loss of lymphatic vessel growth and the authors postulated that post‐MI targeting of lymphangiogenesis may thus fail as a therapeutic modality. However, while this study implies that endogenous lymphangiogenesis mediated by VEGF‐C signalling may be insufficient to ameliorate cardiac function, it does not consider the therapeutic potential of augmenting the endogenous lymphatic response to myocardial ischaemia, i.e. through exogenous means as demonstrated in [48, 50].

## Other factors which promote lymphangiogenesis post‐MI

Adrenomodulin (AM) is a cardioprotective epicardial‐derived factor [89] required for proper cardiovascular and lymphatic network development in mice [90]. AM was increased in response to cardiac injury, including MI [91], and drives lymphangiogenesis via connexin‐43 [92]. Modulation of AM represents a new therapeutic avenue to improve myocardial oedema after cardiac injury.

Apelin plays a key role in lymphatic development, cardiac contractility, angiogenesis, and lymphangiogenesis [93]. After MI, apelin‐knockout mice showed increased VEGF‐C and VEGF‐D with hyperplasia and leaky lymphatic vasculature [94]. Overexpression of apelin was sufficient to restore a functional lymphatic vasculature and to reduce ECM remodelling and inflammation [94]. Furthermore, apelin and the apelin receptor were exclusively expressed on newly formed lymphatic vessels after MI [94].

## Multi‐omic approaches to inform our understanding of neovascularisation and lymphangiogenesis

Advances in high‐throughput single cell/nuclei and spatial omic technologies have transformed our ability to probe the complexity of disease, including MI. The molecular and cellular identities of the heterogeneous cell types that constitute the heart have been thoroughly examined using these technologies in developmental, adult, and diseased states [95, 96, 97]. The single‐cell omics field is rapidly evolving. Genomic, transcriptomic, epigenomic, metabolomic [98], and proteomic approaches continue to undergo exponential scaling in throughput and resolution, accompanied by state‐of‐the‐art tools for in‐depth unbiased integrated analyses and novel target discovery.

Atlases of the healthy human [99, 100, 101] and mouse [82] heart have been generated (Table 2). Unexpectedly, the proportion of ventricular cardiomyocytes was higher in human female hearts and showed a negative correlation with fibroblasts compared with males [100]. However, another study did not report sex‐specific differences in the proportion of atrial and ventricular cardiomyocytes in the human heart [99], which may reflect smaller sample sizes. Furthermore, atrial and ventricular cardiomyocyte populations showed transcriptional differences that indicated different developmental origins and chamber‐specific specialisation [100]. Similarly, distinct transcriptional profiles of atrial and ventricular cardiomyocytes and in particular greater transcriptional differences between the left atrium and ventricle than the right atrium and ventricle were reported, with 2,058 and 1,134 differentially expressed genes, respectively [99]. Genes with unexplored roles in cardiomyocyte function were identified in this chamber‐specific differential analysis [99]. For example, *HAMP*, previously known for iron export activity, was present in 18.3% of right atrium cardiomyocytes compared with other heart chambers. These studies exemplify the power of single nuclear and cell sequencing to gain novel insights into the cellular and transcriptional diversity of the human heart.

**Table 2:** Summary of single‐cell and single nuclei RNA‐sequencing studies in ischaemic heart disease.

Source	Species	Strain	Cell population	Disease	Time point	Method	Type	Number	Published	Reference
LV	Mouse	C57BL/6 J	All	1, 3, 14 days post‐IR1, 14 days post‐sham	8 w	SORT‐seq	SC	2,201	2021	[102]
Whole heart	Mouse	C57BL/6 *N* = 2–4 per time point (time after TAC 0, 2, 5, 8, 11)	All	Healthy and TAC (HF) 0, 2, 5, 8, 11 days	8–10 w	ICELL8	SC	11,492	2020	[103]
LV	Mouse	C57BL/6	CM	Sham and TAC (HF)	8 w	Smart‐seq2	SC	396	2020	[104]
Whole heart	Mouse	C57BL/6JN	All	Healthy	10–15 w	10x Genomics, Smart‐seq2	SC	~5,000	2018	[82]
LV	Mouse	C57BL/6JN	All	Healthy and MI	Adult	10x Genomics	SN	MI: 22,992	2019	[105]
								Healthy: 8,550		
LV	Mouse	Pdgfb‐iCreER^T2^ R26R‐Brainbow2.1	ECs	Healthy and 7 days post‐MI	8–10 w	10x Genomics	SC	28,598	2019	[47]
LV	Mouse	C57BL/6J	All	Healthy and 3 days after IR	8–9 w	SORT‐seq	SC	935	2018	[106]
Ventricles and interventricular septum, excluding cells of the atria, annulus fibrosus, and atrioventricular valves	Mouse	Pdgfra^GFP/+^ mice on C57BL/6J background	Interstitial cells	3 and 7 days post‐sham or MI	8 w	10x Genomics	SC	Sham: 5,723	2019	[107]
								MI day 3: 3,875		
								MI day 7: 3,733		
Whole heart	Mouse	C57BL6/J	ECs	Healthy	8 w	10x Genomics	SC	4,612	2020	[108]
Heart ECs	Mouse	Cdh5‐CreER^T2^; mT/mG	ECs	Homeostasis, days 1, 3, 7, 14, 28 after MI	10–12 w	10x Genomics	SC	15,365	2021	[46]
LV	Mouse	Col1α1‐GFP	Fibroblasts	Healthy 7, 14, 30 days after MI	8–10 w	10x Genomics	SC	29,176	2020	[109]
LV and RV	Mouse	Mki67^TagRFP^ and C57BL/6	Proliferating cells	14 days post‐MI	1 and 8 w	Cel‐Seq2	SC	2,029	2020	[110]
LV and RV	Mouse	Postn^MCM/+^; R26‐eGFP	Myofibroblasts	1 w after MI versus uninjured	8 w	Fluidigm C1	SC	185	2016	[45]
Specific cell type	Mouse	C57BL/6J	Epicardial stromal cells, activated cardiac stromal cells	5 days post‐MI Sham	8–12 w	10x Genomics	SC	Not reported	2021	[111]
Atria and ventricles	Human	N/A	All	Healthy	N/A	10x Genomics	SC and SN	sc45,870/n363,213	2020	[100]
LV	Human	N/A	All	Healthy	N/A	Microwell‐seq	SC	1,308	2020	[112]
								1,478		
Whole heart	Human	N/A	All	Healthy, HF, and recovery	N/A	ICELL8	SC	12,266 (healthy)	2020	[113]
								5,933 (HF)		
Arterial cells	Human	N/A	All	HF	N/A	10x Genomics	SC	125,253	2020	[114]
Whole hearts – 10 μm cryosections	Human	N/A	All	Healthy and MI	N/A	10x Genomics	SN, ATAC‐seq	n191,795/snATAC 46,086	2022	[115]
LV	Human	N/A	All	ICM	N/A	10x Genomics	SC	1,150	2021	[116]
LV	Human	N/A	All	Healthy and DCM	N/A	Smart‐seq2	SC	419	2018	[104]
LVAD cores or identical regions from the apex	Human	N/A	All	Healthy and DCM	N/A	10x Genomics	SC, SN	sc49,723/n 220,752	2022	[117]

CM, cardiomyocyte; DCM, dilated cardiomyopathy; ECs, endothelial cells; HF, heart failure; ICM, ischaemic cardiomyopathy; IR, ischaemia–reperfusion injury; LV, left ventricle; MI, myocardial infarction; N/A, not applicable; RV, right ventricle; SC; single cell; SN, single nuclei; TAC, transverse aortic constriction; w, weeks.

Other sequencing projects have focused on the heterogeneity and dynamics of one cell type, e.g. ECs [47, 108], immune cells [118], and fibroblasts [109]. An analysis of ECs from 11 mouse organs found that EC heterogeneity was predominantly attributed to tissue type, rather than vessel type [108], similar to the Tabula Muris Consortium data that ECs mainly cluster by tissue type of origin [101]. However, capillary ECs had fewer markers that were conserved across tissues, suggesting that capillary ECs show phenotypic variation that is more tissue‐type‐dependent in mice [108]. The authors suggested that this might indicate a greater plasticity of capillary ECs to adapt to tissue microenvironments. In addition, angiogenic and proliferating ECs were identified in the healthy human heart, although in low numbers [108]. Whether this EC phenotype represents a baseline on the spectrum of regeneration requires further examination.

The cardiac EC transcriptome has been examined in ischaemic injury. Single‐cell sequencing of *Pdgfb*‐lineage cardiac VECs from mice revealed heterogeneity in healthy and infarcted hearts [47]. Specifically, ten EC states with distinct expression signatures were reported with predicted functions in proliferation, cardiac and ECM remodelling, among others. Furthermore, a multi‐species meta‐analysis of coronary EC data from scRNA‐seq studies in the healthy and injured mouse and human hearts annotated injury‐associated temporal shifts of the EC transcriptome [119]. Differentially expressed genes in the inflammatory, angiogenic, and vascular maturation phases of MI were identified. This EC meta‐atlas, CrescENDO (http://www.crescendo.science), a searchable app for researchers, exemplifies the value of such data integration.

Recent work implemented a multimodal omics approach using single‐nucleus RNA‐sequencing, single‐cell chromatin accessibility sequencing (snATAC‐seq) and spatial transcriptomics to build a spatial multi‐omic map of human MI [115]. The integration of these data enabled evaluation of cell type compositions at great resolution and identified signatures distinct to pathological sites of injury and remodelling [115]. To exemplify, in ischaemic regions, a reduction in capillary EC proportions was accompanied by an increase in venous ECs. In this way, the expertise of pathologists, scientists, and bioinformaticians can be combined to create essential reference resources for research that give high resolution to pathology. Such approaches will undoubtedly inform the translational research landscape and feed into clinical intervention approaches.

## Clinical strategies for lymphangiogenesis and angiogenesis

Based on the aforementioned pre‐clinical studies and increasing understanding of the innate regenerative mechanisms of cardiac tissue and vasculature [22], there is great opportunity to exploit this new knowledge to develop new effective clinical strategies. Moreover, neovasculogenesis and lymphangiogenesis are plausible targets for therapeutic intervention after MI. A number of different approaches are in development or have been used in clinical studies to treat patients with cardiovascular disease, e.g. administration of stem and progenitor cells, stromal cells, extracellular vesicles and exosomes, growth factors, non‐coding RNAs, episomes, gene therapies, biomaterials, and tissue engineering products [120]. However, to date, most studies have targeted inflammation, infarct size, and modulation of ventricular remodelling. In addition, although most of these studies demonstrated safety in humans, there was significant variability in the efficacy, and significant prolonged clinical improvements were rarely seen. However, inconsistency can be observed in many variables associated with study design and the clinical end‐points evaluated, which may be a source of the conflicting results observed to date.

Strategies for cardiovascular regeneration include administration of exogenous factors, such as cells, implants, grafts, or tissues to stimulate regenerative responses and replace damaged myocardial tissue, and ways to enhance endogenous regenerative responses [120]. Trials modulating the angiogenic response in patients with coronary heart disease, involving VEGF and fibroblast growth factor, have not yet yielded expected cardiac outcomes [121, 122]. VEGF‐A has emerged as a promising angiogenic intervention strategy. The safety of therapeutic angiogenesis was examined in patients with coronary artery disease undergoing surgical revascularisation using *VEGFA*_165_ mRNA therapy. *In vivo* testing showed enhanced blood flow and increased cardiac vascular density in animal studies, and improved cardiac function in pigs undergoing MI [123, 124]. The ongoing EPPICURE phase 2a trial will assess the efficacy of *VEGFA*_165_ mRNA therapy on cardiac angiogenesis.

Despite the technique of *in situ* reconstruction of lymphatic networks using VEGF‐C existing for more than 15 years in pre‐clinical animal studies, the translation of this to clinical trials has been challenging [125]. Pre‐clinical methods of VEGF‐C delivery that improved outcomes post‐MI include implanted particles, recombinant protein, and viral vectors. While viral vectors are established in gene therapy clinical trials, other methods of delivery may be technically challenging to apply to clinical studies [126].

Plasmid‐mediated gene therapy has been a popular delivery method for treating cardiovascular disease [127]. A trial for the percutaneous catheter‐based gene transfer of naked plasmid DNA encoding VEGF2 (phVEGF2) to patients with angina reported a reduction in the Canadian Cardiovascular Society (CCS) angina class and improved the exercise treadmill test (ETT) time [128]. This therapy was also associated with reduced angina in CCS class 3 and 4 patients up to 2 years of follow‐up.

Adenoviral delivery of a pro‐angiogenic and pro‐lymphangiogenic form of VEGF‐D (VEGF‐D^ΔNΔC^) was evaluated for efficacy in patients with refractory angina [129, 130, 131]. Improved myocardial perfusion reserve was reported at 3 and 12 months after administration. However, anti‐adenoviral antibodies increased by 54% in treated patients compared with baseline [132]. In addition, intramyocardial adenoviral VEGF‐D^ΔNΔC^ did not increase the risk for ventricular arrhythmias and may even improve heart rate variability metrics [133]. In a pilot study, intravenous administration of AM was given to patients with acute MI before reperfusion therapy [134]. This showed a significant reduction in infarct size 3 months after treatment compared with baseline.

Indeed, there is a significant paucity of clinical studies that translate the wealth of pre‐clinical data on the benefits of angiogenic and lymphangiogenic modulation into the clinical setting. However, the aforementioned studies provide an optimistic view for angiogenic and lymphangiogenic augmentation after MI as a therapeutic target.

## Conclusion

Most research in cardiac regeneration post‐MI has historically focused on cardiomyocyte proliferation and remuscularisation of the myocardium, although the critical role of both coronary vascular and lymphatic network regeneration is now indisputable. The disappointing correlation of results from pre‐clinical studies to a clinical setting may be mitigated in future by a better understanding of the full repertoire of cellular responses and mechanisms within the complex milieu of the infarcted myocardium, with a particular focus on temporal dynamics. Specifically, delineating co‐regulatory mechanisms, such as those associated with coronary neovascularisation and lymphangiogenesis, may drive the development of more potent therapeutics to target more than one regenerative system. In addition, increasing evidence supports the value of developmental gene pathway reactivation in driving regenerative responses in adult heart disease, and future research in that area may produce significant therapeutic breakthroughs for patients with cardiovascular disease. Finally, the unprecedented evolution of single‐cell and multimodal omics technologies and integrated analysis tools provides a unique opportunity for the cardiovascular research community to align and accelerate target discovery and validation in an unbiased manner. In conclusion, the future of ‘bench‐to‐bedside’ research in cardiovascular regenerative medicine is bright, and holds great promise for the treatment and prevention of HF.

## Author contributions statement

BB, MNHT and MB wrote the manuscript.
